# The utility of the Living Planet Index as a policy tool and for measuring nature recovery

**DOI:** 10.1098/rstb.2023.0207

**Published:** 2025-01-09

**Authors:** Louise McRae, Richard Cornford, Valentina Marconi, Hannah Puleston, Sophie E.H. Ledger, Stefanie Deinet, Philippa Oppenheimer, Mike Hoffmann, Robin Freeman

**Affiliations:** ^1^ Institute of Zoology, Zoological Society of London, London NW1 4RY, UK; ^2^ Genetics, Evolution and Environment, University College London, London WC1E 6BT, UK; ^3^ IIASA, International Institute for Applied Systems Analysis (IIASA), Biodiversity and Natural Resources Program, Laxenburg 2361, Austria; ^4^ Conservation and Policy, Zoological Society of London, London NW1 4RY, UK

**Keywords:** Living Planet Index, biodiversity indicators, Convention on Biological Diversity, biodiversity policy, biodiversity recovery

## Abstract

The Living Planet Index (LPI) is a leading global biodiversity indicator based on vertebrate population time series. Since it was first developed over 25 years ago, the LPI has been widely used to indicate trends in biodiversity globally, primarily reported every two years in the Living Planet Report. Based on relative abundance, a sensitive metric of biodiversity change, the LPI has also been applied as a tool for informing policy and used in assessments for several multilateral conventions and agreements, including the Convention on Biological Diversity 2010 Biodiversity Target and Aichi targets. Here, we outline all current and some potential uses of the LPI as a policy tool and explore the use of the LPI in policy documents to assess the reach of the LPI geographically and over time. We present limitations to the use of this indicator in policy, primarily relating to the development of the index at the national level, and suggest clear pathways to broaden the utility of the LPI and the underlying database for temporal and spatial predictions of biodiversity change. We also provide evidence that the LPI can detect recoveries in biodiversity and suggest its suitability for measuring progress towards the goal of biodiversity recovery by 2050.

This article is part of the discussion meeting issue ‘Bending the curve towards nature recovery: building on Georgina Mace's legacy for a biodiverse future’.

## 1. Introduction

The Living Planet Index (LPI) is a leading global indicator of biodiversity change. It measures the average rate of change in monitored vertebrate populations from a large and growing database of population time series—the Living Planet Database (LPD)—sourced from published literature, grey literature, open databases and contributed data [1,2]. The LPI was conceived as an indicator for biodiversity in 1997 by WWF International [3]. It was initially developed for use as a communication tool to convey global trends in nature to a broad audience through WWF’s biennial Living Planet Report. Work to develop the LPI into a tool for policy started after 2002, when the Convention on Biological Diversity (CBD) set the global target to reduce the rate of biodiversity loss by 2010. The LPI was first highlighted as a potential indicator for the CBD target in 2004 at the Seventh Conference of the Parties [4].

Georgina Mace, to whom this article is dedicated, was one of the organizers of the discussion meeting *Beyond extinction rates: monitoring wild nature for the 2010 target* held at the Royal Society in the following year [5]. There, the LPI was presented to leading academic and non-governmental organization (NGO) researchers working on biodiversity indicators. As a result of this meeting, the first paper on the LPI was published [1]. Georgina, then Director of Science at the Zoological Society of London (ZSL), was interested in the fine-grained picture that can be gained from utilizing population data and saw the LPI as a valuable database and sensitive tool for measuring change over time, particularly with the need for better data and indicators for measuring progress towards the CBD 2010 target [6]. She was instrumental in establishing institutional support for the LPI and securing the LPI as a leading indicator within policy.

The LPI was recommended as an indicator during the meeting of the CBD’s scientific advisory body in 2005 [7], before being adopted for the 2010 target at the Eighth Conference of the Parties later that year [8]. Since 2005, the LPI has become a prominent biodiversity indicator in the policy space. Here, we document its uses to date in international and national policy instruments. We focus on specific applications that exist or could be developed, rather than reporting all applications that may be policy relevant, as these have been catalogued elsewhere [3]. The LPI method has also been used to develop other indicators, including linguistic diversity [9], species awareness [10] and even corporate transparency [11], but we focus here on the LPI itself.

With the recent agreement of the Kunming Montreal Global Biodiversity Framework (hereafter GBF) at the 15th Conference of the Parties (hereafter COP15) to the CBD and the marked absence of a headline indicator for species abundance [12], we also assess the barriers towards the adoption of the LPI, particularly at the national level, and outline other limitations of the LPI as a policy tool. Finally, we outline how the LPI could evolve to meet current and future policy needs and include an example demonstrating how the LPI can detect recovery in biodiversity.

## 2. The Living Planet Index in policy

**Figure 1.**
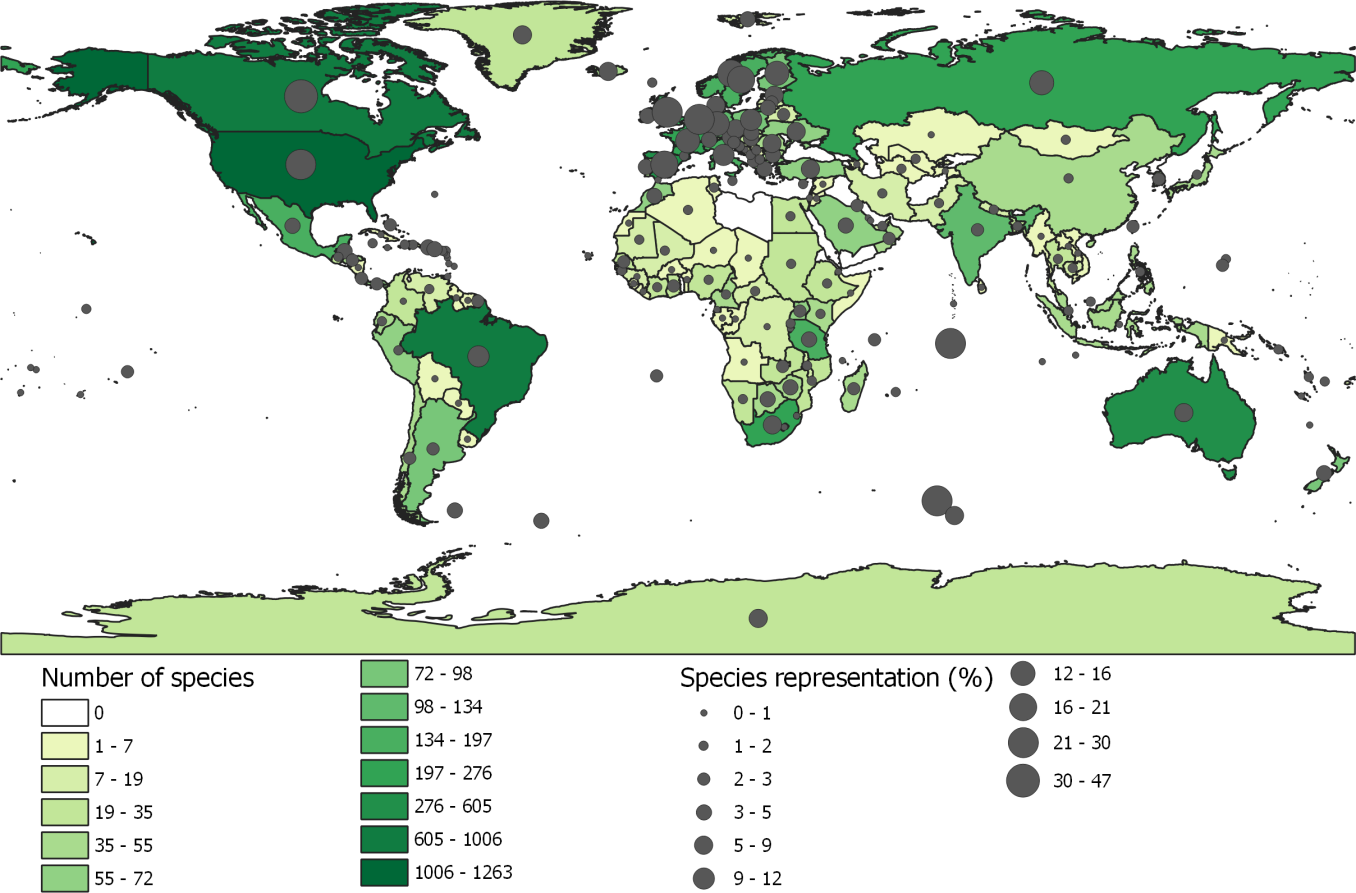
Number of species per country or territory with data available in the Living Planet Database (shading) and the percentage of vertebrate species represented based on national species estimates (circles). Data sourced from https://livingplanet.shinyapps.io/national_map/.

The LPI is a multi-species index calculated using a geometric mean of relative abundance. There are several reasons to use abundance change: species population abundance is considered one of the essential measures of biodiversity [13], the geometric mean of abundance has statistical properties that make it a sensitive measure of change [14,15] and can be considered a form of ‘leading indicator’ for an extinction or as an extinction risk target because it shows a response at the first sign of changes in the subject being measured, i.e. declines in population abundance are precursors to changes at the species level leading to extirpations and extinctions [16]. In addition, goal A of the recently agreed GBF explicitly includes a clause referring to wild species' abundance [17]. Indeed, multi-species indices are widely used for indicating changes in biodiversity at different scales, such as the UK and the Netherlands [18,19], and for specific taxonomic groups, e.g. Wild Bird index [20]. While each have their own specific statistical approaches, they all aggregate species population trends to produce an average rate of change among the populations and species included. A notable difference between the LPI and other multi-species indices is that the latter are largely based on species abundance data on a range of taxa collected as part of national monitoring schemes [18,20], whereas the LPI is constructed from a broad range of data sources for vertebrate species around the world that vary in timeframe and scale [3,21]. The reliance on publicly available data through scientific papers and online databases renders the index subject to bias present in the scientific literature. This results in a disparity in how well different taxa and regions are represented and is arguably the main limiting factor to the robustness of any application of the LPI, particularly for regions other than Western Europe and North America (figure 1) and taxa other than mammals and birds [3]. For instance, two examples of the regular use of the LPI at the national and sub-global scale—Canada and the Arctic (see §2a)—are for regions that benefit from a steady input of standardized abundance data delivered by long-term monitoring schemes. However, well-designed and recurrent monitoring programmes for species are scarce across many regions of the world [22], which limits the availability of data and results in the uneven representation of species and regions in the LPI. At the global level, a weighted approach was introduced to the current LPI method that places proportional weight on trends according to how species rich a taxa and biogeographic realm is [23]. While it is not a perfect solution (see [3,24]), the weighting is designed to improve the index over an unweighted version [23].

While data can be a limitation, there are a number of other characteristics that make the LPI potentially suitable for application to a range of policy contexts including cost-effectiveness, scalability and frequency of reporting [25] and it theoretically meets all of the criteria of a ‘successful’ indicator for national development [26], although this has not translated into broad uptake by countries because many other factors are at play (as discussed in §3). Data, peer-reviewed methods and code to calculate an LPI are freely available online alongside a guidance document for developing national applications [27]. In addition to population data, the LPD contains multiple fields on species ecology, population threats and management, alongside site-level information, enabling the LPI to be tailored to many different policies. These factors, along with the high profile of the LPI as the headline statistic in the Living Planet Report, likely explain the frequency with which the LPI has been used in policy to date.

### (a) Applications of the LPI in policy

Within international policy, the LPI has primarily been applied to measure progress towards CBD targets. It was one of a suite of indicators used to evaluate the achievement of the 2010 target [28] and subsequently, the Aichi targets as part of the 2011–2020 strategic plan for biodiversity [29–31]. Under the GBF, indicators are listed in the monitoring framework as a headline (high-level indicators recommended for use by parties to measure progress towards goals and targets), component (these are optional and measure components of the goals and targets) or complementary indicators (these are optional and measure a thematic element of goals and targets). The global LPI is listed in the current framework as a component indicator for goal A and target 4, and a complementary indicator for target 2. The LPI for utilized species—a disaggregation of the LPI [32]—is a component indicator for goal B, targets 5 and 9; and the growth in the number of records and species in the LPD is listed as a complementary indicator for target 21 (table 1 [17]).

**Table 1.** Current and potential uses of the LPI across the major multilateral agreements and conventions with examples where available. These examples can be developed at a global or national scale. Adapted from Ledger *et al.* [3] with additional information on indicator mapping [33,34]. CBD GBF: Convention on Biological Diversity Global Biodiversity Framework, SDG: Sustainable Development Goals, CMS: Convention on Migratory Species, Ramsar: Ramsar Convention, CITES: Convention on International Trade in Endangered Species, IPBES: Intergovernmental Panel on Biodiversity and Ecosystem Services, UNCCD: United Nations Framework Convention on Climate Change.

		Biodiversity and sustainable development targets and other multilateral environmental agreements							
**themes**	**examples**	**CBD GBF**		**SDG**	**CMS**	**Ramsar**	**CITES**	**IPBES**	**UNCCD**
		goals	targets						
**global**									
global	Living Planet Index [23,31,35]	A	4	14,15				✓	
**ecosystem**									
freshwater	Living Planet Index (freshwater populations) [35,36]	A	2	15		✓			
wetlands	Living Planet Index (estuarine populations) [37] Living Planet Index (wetland populations) [38,39]	A	2	15		✓			
marine	Living Planet Index (marine populations) [40]	A and B	2 and 10	14					
terrestrial	Living Planet Index (terrestrial populations) [23,41]	A	2	15					
drylands	Living Planet Index (drylands) [42]	A	2	15					✓
forests	Living Planet Index (forest specialists) [43]	A	2	15					
**ecology**									
migratory species	Living Planet Index (migratory species) [44–46]	A	2	14,15	✓				
	Living Planet Index (migratory freshwater fish) [47]	A	2	15	✓	✓			
**drivers and conservation management**									
conservation action	Living Planet Index (populations under conservation management) [48]	A and B	4	15					
	Living Planet Index (protected areas) [49]	A	2	15		✓			
species in use	Living Planet Index (utilized populations) [30,32]	B	5 and 9	12			✓	✓	
	Living Planet Index (populations impacted by fishing or bycatch) [40,50]	B	5 and 9	14				✓	
climate change	Living Planet Index (species vulnerable to climate change)		8	13					
**knowledge and data**									
availability of biodiversity data	growth in the number of records and species in the Living Planet Database		21						

Among other United Nations (UN) conventions, an LPI for species that migrate has been included in two status reports for the Convention on Migratory Species (CMS) in 2008 and 2019 [44,46] and the LPI is one of the indicators in the current CMS strategic plan (table 1; [51]). Since 2002, the LPI has been featured in eight UN publications on the state of biodiversity: four editions of both the Global Biodiversity Outlook [31] and the Global Environment Outlook [52]. The first scientific assessment of ecosystems, the Millennium Ecosystem Assessment, also included the global, terrestrial, freshwater and marine LPIs [53]. The LPI is not currently one of the indicators adopted for monitoring progress towards the Sustainable Development Goals (SDGs). It was proposed as a tier II indicator to complement the Red List Index (RLI) for goals 14 and 15 as part of the 2020 comprehensive review [54], but ostensibly the lack of readily available LPIs at the country level meant that it was not accepted (see §3).

Outside of UN processes, the LPI is featured within the Intergovernmental Panel on Biodiversity and Ecosystem Services (IPBES) framework [55] and has been used in the global assessment of biodiversity and ecosystem services [56] and the regional assessment for Europe and Central Asia [57]. The lack of a species indicator for tracking the impact of utilization had been identified by the IPBES as a priority gap to fill [58] and this had also been recognized as a requirement in the CBD monitoring framework. In response, a new disaggregation of the LPI was developed to monitor trends in utilized populations [32]; as well as presenting a new indicator, this work revealed that populations under targeted management fared better than unmanaged ones [32]. These new global and regional LPIs for utilized populations were included in the status and trends chapter of the IPBES Thematic Assessment Report on the Sustainable Use of Wild Species, and the evidence for the positive impact of management was also highlighted in a section on sustainable use [59].

The disaggregation of the global LPI into thematic or regional indicators has broadened its application among other conventions and international bodies. For example, an ecosystem approach using LPIs for terrestrial, freshwater and marine populations highlighted that the freshwater index was in steepest decline as part of the Ramsar Convention reporting on trends in wetland biodiversity [36]; the LPI has since been identified as a suitable indicator for monitoring trends in wetland biodiversity (table 1; [33]). An LPI for species identified as a forest specialist (Forest Specialist Index) was developed to shed light on species trends in forest ecosystems and to fill a gap in the set of forest indicators that are largely based on habitat metrics [43]. The Forest Specialist Index was included as part of a section on policy targets relating to forest species in the Food and Agriculture Organization (FAO)’s 'State of the World’s Forests 2020' [60] and provided a vertebrate population trend metric for forests within global biodiversity frameworks, where measures of forest area and extent currently dominate.

At a sub-global level, the LPI was used to show trends in marine vertebrates in Europe by the European Environment Agency [61]. The Arctic Species Trend Index and Arctic Migratory Birds Index are LPIs developed for the Conservation of Arctic Flora and Fauna (CAFF), the biodiversity working group of the Arctic Council [62,63]. These indices were designed to measure biodiversity change within both the ecological and political boundaries of the Arctic; the Migratory Bird Index also incorporated data from outside the Arctic for species identified as Arctic migrants. The Arctic Species Trend Index was included in the 2013 Arctic Biodiversity Assessment for the Arctic Council [64] and used to monitor progress towards the CBD Aichi targets.

Multi-species indices akin to the LPI have been developed for use by countries, particularly where long-term monitoring schemes are in place. For example, abundance indicators have been published for the Netherlands [19], Belgium [65], England [66] and Scotland [67], with vertebrate, invertebrate and plant species included in the latter. The first national LPI was produced for Uganda in 2004 by Makerere University, which published this and several further reports on the State of Uganda’s Biodiversity [68–72]. The 2004 ‘Living Uganda Index’ was presented as a case study to illustrate how the LPI could be used as a tool to track biodiversity at a national scale at the Seventh Conference of the Parties of the CBD [4]. The Canadian Species Index (CSI), an LPI for Canada, was the first indicator to be produced in response to a national policy need and has been used as part of the Government of Canada’s suite of environmental indicators since 2014 [21,73]. These approaches demonstrate the flexibility of the LPI method to different national contexts and the robustness to small methodological changes; for example, the CSI was adapted from the global LPI to model short time series and transform zero values in a slightly different way [21]. Species monitoring in Canada has been extensive enough to provide a dataset that represents over half of native and regularly occurring Canadian vertebrate species [21]. Although gaps remain in the coverage of vertebrate species and representation of life-history traits [74], the CSI is still the best-represented national dataset within the LPI (electronic supplementary material, table S1).

Another adaptation to the LPI approach was taken by the Threatened Species Recovery Hub in Australia, which developed the Threatened Species Index [75]. This indicator uses the LPI method for measuring trends in threatened and near-threatened species of Australia at national, state and regional levels. The Threatened Species Index was originally developed to monitor progress towards Aichi target 12 (extinctions prevented) and was recently adopted by the Treasury of the Australian Government as the key biodiversity indicator for Australia’s first Wellbeing Framework [76]. It is also an example of how a national LPI can be expanded taxonomically, as the Threatened Species Index was the first adaptation of the LPI method to plants [77]. The Threatened Species Index is now managed by the government-funded Terrestrial Ecosystem Research Network and plans regular updates to the indicator from long-term monitoring programmes across the country.

To facilitate the production of national-level LPIs, guidelines have been produced to provide a reference for their development, allowing governments, NGOs and research organizations to calculate an index for their country [27]. Several countries, including the Netherlands, Uganda, Canada and China, have included national species abundance indicators in their national reports to the CBD, but the LPI has not yet been widely adopted at the national scale in policy, for reasons we discuss below.

### (b) How the LPI has been cited in policy documents

To explore the impact of the LPI in policy, we searched the Overton policy database [78], the largest global repository of policy documents available, for documents published by and for policymakers since 2015 and containing the search term ‘Living Planet Index’ in English and in all six official languages of the CBD (see electronic supplementary material, Methods). We excluded documents authored by WWF and summarized the remaining documents to explore trends over time, the source type (Government, Think Tank, Intergovernmental Organization (IGO)) and the country where they were published. There were 483 policy documents resulting from the English language search and 513 policy documents from the multilingual search. Of the 513, 171 were categorized as government policy documents, 223 from IGO sources and 108 from think tanks; a larger proportion of documents from this search were from IGO and think tank sources compared to policy documents within the topic of biodiversity as a whole (electronic supplementary material, figure S1). Since 2015, at least 28 publications annually have cited the LPI (electronic supplementary material, figure S2). The peak of 94 policy documents in 2019 is likely linked to the post-2020 discussions of the CBD. The temporal pattern of documents within the topic of biodiversity shows a gradual increase up to 2021 and, as mirrored in the LPI search, there are fewer documents available for recent years (electronic supplementary material, figure S2).

Documents were published by sources from 32 countries, with most coming from North America and Europe (electronic supplementary material, figure S3). In the same database, we further explored how these 513 policy documents have been cited since 2015 and found them referenced within 4699 documents covering 64 countries, indicating a broader global reach (electronic supplementary material, figure S3). We benchmarked the LPI results in English only to the search term ‘Red List Index’, the headline indicator for species targets and goals in the GBF. After removing results published by the IUCN, we found 754 policy documents and there was a similar temporal trend to the LPI results, but the documents that cite the Red List Index came from 54 countries and show a broader global spread, particularly within the African and Asian regions.

These results need to be interpreted with some caution since the Overton database does have limitations in temporal coverage pre-2015 and has a geographic bias towards Europe and North America [78]. A language bias has not been quantified and although there are 66 languages included and good coverage of local languages compared to bibliometric databases, the English language dominates perhaps in part owing to the nature of the geographic bias [78,79]. However, the Overton database does give a snapshot of the regular use of the LPI in global and national documents and highlights regions of the world, namely Africa and Asia, where it has less traction. Overall, the LPI has been cited in fewer policy documents than the Red List Index. This likely reflects the fact that the RLI is a key indicator for more multilateral environmental agreements than the LPI (the SDGs, IPBES); it is supported by an Intergovernmental Organization (IUCN) that itself produces many key policy documents that reference the LPI (there were 28 IUCN-authored documents in the LPI search on Overton); and crucially, it is readily available at the national level, either as a disaggregated global indicator or from national data directly, although it has still not had significant uptake by countries for their CBD national reports [80]. Country-level LPIs, by comparison, are only available for a handful of nations, and this is likely one explanation behind its lower frequency of citation in government policy documents.

## 3. Limitations of the LPI as a policy tool

Ledger *et al*. [3] have previously summarized some of the main challenges to the use or adoption of biodiversity indicators, including the LPI, such as the sensitivity of the method to extreme trends, data availability, and the way they are communicated. Some technical limitations are common to multi-species indices, such as the sensitivity of the geometric mean to outliers [20], zero values [81,82] and rare species [82,83]. Furthermore, uncertainty from both measurement of the population and stochasticity within a population tends to bias a geometric mean downwards [81,84], and this is especially pronounced when population trends covary [85]. Other sensitivities detected in the LPI relate to the unevenness in temporal coverage found within the dataset, where short or sparse time series tend towards declining trends but also demonstrate higher uncertainty in the trend estimates [81,86]. Variation in trends in the LPI is captured by bootstrapping the species trends but this does not represent uncertainty in the underlying population estimates or trends. A recent power analysis using LPI data found around 20% of country-level subsets assessed had a 70% or greater certainty of showing a strong decline, meaning there was high uncertainty of declines in the remaining dataset [87]. When incorporating spatial, temporal and phylogenetic structures, the high level of uncertainty in the LPI data and nine other time series datasets was such that no significant trends were detectable [88]. While methodological developments can address some of these limitations (see §4), the previous two examples highlight uncertainty within the dataset itself and emphasize the ongoing constraint of data availability.

One of the main barriers in the application of the LPI for policy at the national level pertains to both a real and a perceived constraint around data and resources. Real, because in some cases the data simply do not exist and considerable resources would need to be invested to generate these. Perceived, because it is possible to produce even a preliminary, yet informative, LPI with relatively limited data provided those species data are good quality and representative. Currently, the LPI database contains data from 202 countries and territories, although the number and coverage of vertebrate species vary (figure 1; electronic supplementary material, table S1), meaning that for many countries there may be insufficient data to calculate a representative national LPI without additional mobilization of data. It is difficult to establish a minimum threshold of data needed for a country because this depends on the national context and representation of the species for which data are available. As a baseline, there are data for over 50 species from over 40 countries in the LPI database, but an assessment is needed for each to confirm whether this is a sufficient starting point for any given national LPI. This limitation is a primary reason why the LPI is not an adopted indicator for the SDGs or a headline indicator in the GBF. In a technical workshop held in advance of the GBF negotiations at the COP15 to score indicators as to their headline indicator suitability, the LPI was given a score of 2, which denoted ‘Support for inclusion as a headline indicator but does not currently meet all the assessment criteria and further development necessary’. The workshop also highlighted that the LPI had high-capacity needs for national development, suggesting that it is not only data that are a limiting factor but also the necessary tools and resources to build the indicator [89].

In reality, a national LPI can be relatively cost-effective to develop and maintain since it relies on available monitoring data [25,90], much of which is often already published but not mobilized. Mobilizing and processing data do require capacity, and can be difficult to access [91], but even low-level efforts to increase coverage can yield data quickly, especially when focused on sources published in languages other than English, which form a substantial part of biodiversity literature [92]. For example, in the 2022 update of the global LPI, searches in Portuguese have rapidly expanded the dataset for Brazil, trebling the number of species represented to over 1000 in a few months [35]. Other approaches such as automating data searches have been identified as opportunities for expanding geographic and taxonomic coverage of LPI data [3].

Finally, the LPI has benefitted from long-term institutional support from the current partners WWF UK and the Zoological Society of London, which is one of the key factors identified for indicator uptake by the Biodiversity Indicators Partnership [26]. That the LPI’s development is overseen by these NGOs based in Europe, and not by an IGO, could be a barrier to national uptake, particularly as data ownership can be a critical factor in how biodiversity information is used [93]. Some agreements such as the SDGs require their indicators to have a recognized custodian agency such as a UN body. These intergovernmental organizations tend to have a greater global reach and institutional capacity but may be constrained in other ways. Ultimately, there are other factors tangential to the qualities of an indicator that influence whether countries use them for their national reporting; even the Red List Index, which is available in disaggregated form at the country level, has had low uptake by parties with many preferring to develop versions more relevant to their national context [80].

## 4. Meeting current and future policy needs

The LPI needs to evolve in step with the policy landscape and methodological developments to remain a key biodiversity indicator and resource at the global, sub-global and national levels. Here, we outline current opportunities for the use of the LPI in policy and identify which barriers to its adoption should be urgently addressed, alongside some methodological considerations for meeting future policy needs.

### (a) Methodological development

The LPI has been designed to measure changes in vertebrate populations that have occurred within the locations monitored and up until the recent past or when the most recent data are available. We propose four methodological developments that could increase the utility of the LPI in policy at all scales. First, continual development of the LPI method should be explored to reflect advances in modelling since the last change to the statistical framework [2]. For example, Bayesian models can be applied to capture uncertainty within the population observations but also in the aggregated trends, providing an estimate for the LPI that enables the reliability of the underlying data to also be communicated. Such models have already been applied to the LPI data [87,94,95]. Given the concerns raised around the robustness of some of the national and regional trends in the LPI [86,87], a clear measure of uncertainty would help in providing a transparent and robust report of the LPI for policymakers.

Second, developing explanatory or causal models using LPI data alongside environmental and driver data could underpin a number of applications with uses for policy. Sophisticated models have already been built that incorporate land-use change, climate change, utilization, management and protected area variables to model lagged responses in terrestrial bird and mammal populations [96]. These could be extended to include pressures such as pollution and invasive species and other taxonomic groups, or for using counterfactual approaches to understand causal mechanisms. Ongoing efforts to mobilize and collect new population time series could continually add empirical data into these models, which become better informed over time. Such models can be used to spatially extrapolate trends for a country and generate a predicted national LPI, which is particularly beneficial where limited data are available. A predicted national LPI may have high uncertainty that would need to be visualized and communicated clearly to inform policymakers of the reliability of the estimated trends. Countries or areas with high uncertainty could then be used to identify new priorities for species monitoring.

Models quantifying the relationships between different drivers and population responses could also form the basis of temporal predictions of the LPI under different projected rates of environmental change. The terrestrial LPI has been applied in this way to show how the index might respond to projected changes in land-use under different scenarios of food production and consumption, combined with conservation management; results suggest that an integrated portfolio that reduces the pressures from food production and consumption, combined with increased conservation, is needed to reverse biodiversity declines [41]. Building on this approach by incorporating other environmental drivers, such as climate change, would allow the LPI to evolve from describing a state or recent trends to making predictions for the future under alternative scenario models [97].

Third, identifying how an indicator responds as it moves towards a set target can help identify whether conservation policies and actions are working [98,99]. This will vary from indicator to indicator depending on the underlying metric [98,99]. Providing policymakers with an indicator that can illustrate how far away this trajectory is from a set target could provide evidence on the level of ambition required to meet the target. For example, the Forest Declaration Assessment includes indicators that show how global and regional rates of forest loss compare against targets to reduce rates of deforestation and where trends are off track for meeting the targets [100]. England has set a legally binding target for halting abundance decline in the short term, and increasing species abundance by 10% in the longer term to be assessed by an LPI-like indicator including vertebrates, invertebrates and plants [66,101,102]. In contrast, goal A of the GBF refers to an increase in wild species abundance to healthy and resilient levels but does not contain a quantitative target. Given the uncertainty identified in subsets of the LPI trends [87], work is needed to formulate what an on-track trend in abundance (LPI or otherwise) should look like to effectively and precisely measure progress towards these quantitative targets.

### (b) Mobilizing and accessing data

The lack of representative data at the national level has been identified as one of the fundamental blockages to the use of global biodiversity data by countries [93]. Although the LPD is available online, it may not be widely known, so increasing awareness is an essential first step in enabling the use of national data [93]. However, data will still need to be mobilized for countries and this is best implemented by organizations within the country in question to better capitalize on knowledge of monitoring schemes, capturing data published in local languages and ensuring appropriate ownership and control of the national dataset [92,93,103]. A review of indicator use within the fifth national report for the CBD found that the uptake of global indicators and those recommended by the CBD was low, with a preference for nationally generated indicators [104]; this is the case also for disaggregated national RLIs, which have rarely been used in national reports [80], and suggests that data availability is not the only factor determining the uptake of global indicators at the national level. The LPD could still be a useful resource for generating other indicators for countries even if the LPI is not the method of choice; as noted above, the number of records and species in the LPD is a complementary indicator for target 21 of the GBF. The LPD could also serve to highlight current gaps in data that have been mobilized from the scientific literature, and the LPI data search protocols and tools for analysis are available for guidance.

Actions have been identified to increase the availability and usability of species abundance data and biodiversity information in general [3,93,103], many of which could help to increase the application and content of the LPD. The LPD is biased towards well-studied species and regions, which is driven by the associated bias in species monitoring [22,23]. Taxonomic groups and regions with lower availability of data often demonstrate greater uncertainty in the resulting trends, and increasing monitoring efforts as well as mobilizing existing data have been recommended as approaches to improve the power to detect trends [86,87]. While greater support for monitoring is needed, alongside approaches to improve survey design and implementation [12,91], these will take time to yield useful information; however, efforts to mobilize existing data could quickly increase data availability. Searches for data to include in the LPI have been primarily conducted in English [3] and as noted earlier, searching in other languages can rapidly augment a dataset for a given country. This could be enhanced by using predictive models trained in multiple languages to automate searches, reducing the bottlenecks associated with manual data searches [105]; open-source large language tools such as ChatGPT could also be harnessed to do this, although their models are optimized for English and are likely to be more effective for languages with large available data resources [106].

### (c) Potential applications

In addition to current applications, a portfolio of global thematic LPIs could be applied for monitoring towards other conventions (table 1). For example, the LPI has been used to measure trends in species that live in drylands [42], providing a measure of ecosystem health that could be relevant to the UNCCD (United Nations Convention to Combat Desertification). The interconnected relationships between climate and biodiversity have been given new emphasis within the UNFCCC (United Nations Framework Convention on Climate Change). Although indicators for the UNFCCC focus on essential climate variables, biodiversity indicators are increasingly needed to demonstrate both the impacts of climate change on species and to monitor recoveries in biodiversity (target 8 of the GBF and target 13 of the SDGs), as improved ecosystem functioning is one of the solutions needed for mitigating climate change. The LPI could play a role in tracking trends in particular ecosystems, such as wetlands and forests, which are key to implementing these nature-based solutions or for species identified as vulnerable to climate change. Tracking the latitudinal or regional impacts of climate change could also be done using subsets of the data. As well as monitoring trends for a relevant habitat or taxa, the LPI can also show populations to specific drivers of change, such as invasive species, illegal wildlife trade and overfishing. This increases the potential applications of the LPI to several targets within goals 14 and 15 of the SDGs and, although the status and trends in the Convention on International Trade in Endangered Species (CITES)-listed species are not among the goals and objectives of the CITES strategic plan, the LPI can give a broad picture of trends in these species, noting that drivers other than trade may be influencing them (table 1).

Negotiations at COP15 culminated in the significant inclusion of species abundance within goal A, with the aim to increase abundance to healthy and resilient levels; however, a headline indicator for abundance is lacking within the monitoring framework [12]. The LPI is an ideal metric (it is listed as a component indicator for goal A), and arguably the index and database could provide the foundation for countries to use as a headline indicator within the GBF if support for species monitoring at a national level is increased [12]. With the emphasis on the GBF and other policies such as the European Union Nature Restoration Bill and the UK Environment Act on Recovery and Restoration, indicators will need to robustly detect positive changes in trend. Where quantitative targets are set, as is the case for species abundance in England, indicators must track progress towards such targets, measure the precision of the trends and, importantly, understand the many possible mechanisms, from large recoveries in a few species to small recoveries in many, by which a target could be achieved [66].

## 5. How the LPI could detect recovery at the global scale

**Figure 2.**
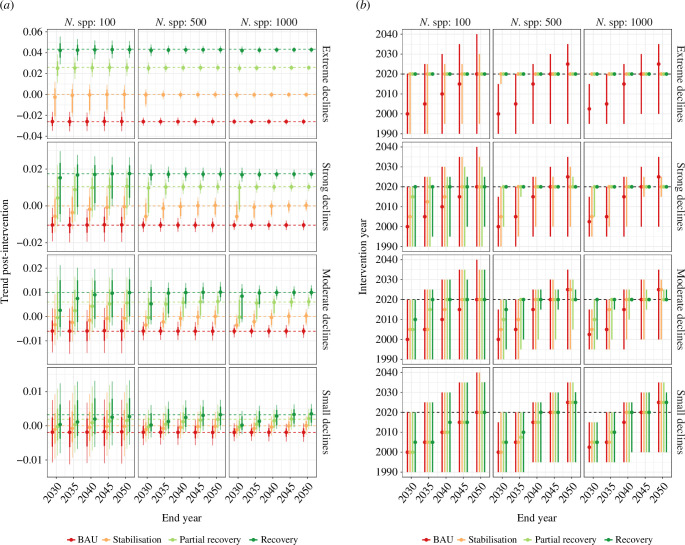
Results of the linear mixed-effects models showing the distribution of estimates across the 5 end years for the slope after the intervention (*a*) and the intervention year (*b*). Points indicate the median value across simulations, thick bars show the 95% inter-quantile range of the coefficients and thin bars show the 95% inter-quantile range of coefficient confidence intervals. The distance between the points and dashed lines (closer = more accurate = better; colour coordinated in (*a*), in addition to the size of the error bars (smaller = less uncertainty = better), indicates the ability of the models to estimate the values of interest. Columns present results based on simulations with different numbers of species, and rows the different assumptions about historic declines. Colours depict the future scenarios considered. Note: in the BAU (business as usual) scenario, there is no intervention year.

Here, we present an alternative approach using simulated data to explore whether the LPI could detect changes in trends under three trajectories of recovery from different baselines. To retain relevance as a global (and national) biodiversity indicator, an abundance index like the LPI should be able to detect changes in population trends that might signal recovery in biodiversity. Using simulated data, which was sampled to more realistically reflect the imperfect properties of data contributing to the LPI, we quantitatively explored how accurately the impact and timing of such interventions could be detected (see electronic supplementary material, Methods). We explored four different trajectories of historical trends that broadly represent the regional trends in the LPI between 1970 and 2020 (electronic supplementary material, figure S4; extreme declines: −95%, strong declines: −70%, moderate declines: −50% and small declines: −20%) and four different scenarios of future trends (2020–2050, figure 2*a,b*, electronic supplementary material, figure S4; business as usual, stabilization, partial-recovery and recovery). We present the distributions of model-estimated values for post-intervention population trends (figure 2*a*) and the intervention year (figure 2*b*) for each of the four historical and future trends, and for three datasets of different sizes. We found that the post-intervention trend could be estimated accurately in most scenarios, particularly with datasets of more than 500 species/populations and even over short future time scales (10 years) when historical declines and subsequent recoveries were strongly contrasting (figure 2*a*). As expected, post-intervention trends (recovery, partial recovery and stabilization) were detected more accurately and earlier for larger historical declines (figure 2*a*).

The intervention date of the ‘recovery’ scenario (return to the 1970 baseline between 2020 and 2050) was accurately detected in all cases except for smaller historical declines (figure 2*b*). Intervention date under partial recovery was accurately detectable for strong and moderate historical declines (with at least 500 populations per species). Under stabilization, the date of intervention was usually detectable for extreme and strong declines. These initial results suggest that the LPI can detect recoveries in aggregated time series using simulated data—even with time series of varying lengths and fullness—over a period of at least ten years. We note that while we are able here to quantify the confidence of the estimated change in trends and its change point, we have not built in any variation in population uncertainty across taxonomic groups or regions that we know exists in real data. This is a caveat, particularly in the context of recent results demonstrating that uncertainty in recent trends (e.g. over a 7-year span) in subsets of the LPI limits the detection of future changes in trend [87]. Future work might usefully extend this approach by mirroring the taxonomic and geographical biases in real datasets; this will be critical to assess the ability of the LPI to detect significant changes in trends— especially where uncertainty is high, such as within data-poor subsets.

## 6. Conclusion

The LPI has been one of the key biodiversity indicators for global policy since 2005. It was used to demonstrate the failure to meet both the CBD 2010 and Aichi targets and has been applied to monitoring frameworks for other conventions, as well as at the national scale in some instances. The priorities for the development of the LPI are to improve the representation of data to enable the development of national LPIs. The next generation of methods for calculating the LPI should review the current approaches to modelling and aggregating the data, and pioneer the work on extrapolating and predicting trends in vertebrate populations, spatially and temporally. This is reliant on further developing models linking drivers and trends so that environmental variables and scenarios of land-use and climate change can be used to estimate LPI values. Modelling extrapolations of the LPI at the national scale could serve as a valuable tool for measuring a country’s progress towards biodiversity targets. Simulated results demonstrate that the LPI can detect the timing of recovery in abundance across a range of potential scenarios. If we hope to detect the recovery of species abundance to healthy and resilient levels, the ability to quantify such changes will be critical to assessing future progress.

## Data Availability

For figure 1, we used data from Living Planet Database that can be downloaded from https://www.livingplanetindex.org/data_portal or see the summary that we present at https://livingplanet.shinyapps.io/national_map/. For figure 2, we used simulated data. For the analysis of the LPI in policy documents, we used the Overton database (see https://www.overton.io/). This is not publicly accessible except through a free trial. The link to the report from the LPI search can be viewed in Supplementary material, available online [107].
